# Hormones can influence drug addiction-A narrative review

**DOI:** 10.37796/2211-8039.1120

**Published:** 2021-06-01

**Authors:** Hamidreza Famitafreshi, Morteza Karimian

**Affiliations:** a Physiology Department, Tehran University of Medical Sciences-International Campus, Tehran, Iran; b Physiology Department, Tehran University of Medical Sciences, Tehran, Iran

**Keywords:** Addiction, Withdrawal, Stress, Sex, Hormones, Thyroid, Neurohypophyseal

## Abstract

Drug addiction is a dangerous condition that is the concern of human societies. Nevertheless, several issues exist ahead of people with a drug use disorder during addiction. Accordingly, various types of studies have been conducted to understand better the problems that people with a drug use disorder encountered. People with a drug use disorder usually have a problem tolerating the withdrawal period and some relapse to drug abuse. Complementary studies further revealed that some hormones like oxytocin (OXT), vasopressin, hypothalamic-pituitary-adrenal axis (HPA) axis hormones, sex hormones, thyroid hormones, and nutritional hormones are probably involved in addiction processes. These hormones completely with different mechanisms can influence drug users’ ultimate outcome. As we will see, these hormones influence tolerance, sensitization, and compulsive drug seeking and taking behavior. In this review, they have been further discussed.

## 1. Introduction

Drug addiction is a severe socio-economical problem and plenty of studies have been done to reveal the complex aspects of this problem [1]. The foremost downside that people with a drug use disorder encounter is to progression to uncontrolled use of those substances [2]. The monoaminergic and cholinergic systems are the most important systems for investigating these types of behaviors. In different sets of studies especially with cocaine in human subjects, these brain systems have been shown to be important for modulation of some important neuronal pathways. These are the coeruleo-cortical noradrenergic system that regulates divided and selective attention, the basal forebrain cholinergic system that is important for stimulus detection, and the mesostriatal and mesolimbic dopaminergic systems that are important in reward-regulated behaviors. Psychotropic drugs make some changes in the reward circuit and perhaps another alternative part of the brain that renders individuals to develop full manifestation of uncontrolled addiction [3]. These modifications are the changes in the degree of tolerance, the emergence of sensitization, and changing the affinity of drug-receptors. Accordingly, some hormones such as neurohypophyseal hormones and sex hormones have recently been shown in different studies both in animals and human subjects which will interfere with reward circuit neurons and exert some changes which will make prone individuals with a dangerous addiction. Recent studies recommend that Neurohypophyseal hormones like oxytocin and vasopressin [4], stress hormones [5], and sex hormones [6] will have an affect reward circuit that has been observed in animal and human studies. These hormones can change tolerance, sensitization, and self-administration. It should be noted that study models for investigating behaviors such as sensitization and self-administration are mostly assessed by animal studies and in human studies are sparse [7]. Here we are going to reveal the different aspects of these hormones within the addiction process.

## 2. Neurohypophyseal hormones

The shreds of evidence that support the role of Neurohypophyseal hormones in addiction come from animal studies. After subcutaneously, orally and intracerebroventricularly administration of desglycinamide^9^-vasopressin (DG-AVP), heroin self-administration in rats was reduced [8]. This action is dependent on the sub-regions of the molecular structure of these hormones because the C-terminal tripeptide of oxytocin (OXT), prolyl-leucyl-glycinamide (PLG) facilitates the acquisition of self-administration [9]. In a recent study, it has been postulated that alcohol and psychostimulant interfere with vasopressin action on the brain and will result in behavioral abnormalities [10]. Later studies with morphine brought convincing evidence that OXT could interfere with addictive behavior via the reward circuit. The most sensitive areas of the brain are the hippocampus and basal forebrain [11, 12]. The primary proof which shows OXT interacts with the mesolimbic dopaminergic system comes from analgesic treatments with morphine in albino mice [13]. However, later studies suggest that this impact can also be seen with the administration of non-analgesic doses in animal studies [4]. Also in the presence of nicotine dependence and the emergence of withdrawal signs, after administration of OXT, the severity of withdrawal signs reduces [14]. A study with heroin showed that the primary action of OXT is not reinforcing properties of heroin, but rather on the degree of tolerance and dependence on heroin in rat models [15]. In a recent study with alcohol, OXT blocks enhanced motivation for alcohol abuse through GABAergic transmission in the central amygdala rat model of alcohol dependence [16]. Despite the presence of OXT receptors on limbic areas, it has been suggested that OXT may exert its effects through vasopressin receptors in the brain of male, adult rats [17]. Also, there are pieces of evidence that propose OXT may partly influence addictive behavior through dopamine receptors in the basal forebrain of the mouse [18]. In alternative complementary studies with cocaine and ethanol, it was shown that OXT can interfere with locomotor activity, tolerance, and sensitization in mice models [19, 20]. Intranasal treatment of OXT has been suggested for clinical trials and clinical trials for OXT are ongoing as there are many trials in the clinicaltrial.gov database for OXT and different drugs such as alcohol, nicotine, and morphine, but vasopressin in a controlled study showed promising effects for the facilitation of methadone therapy in a clinical trial in human subjects [21].

## 3. Stress hormones

Psychotropic drugs are by themselves are potent stimulators of stress hormone release. The strong evidence for the role of addictive drugs as stimulators of stress hormone release comes from an animal study that showed by a single injection of a bolus dose of Alcohol, the HPA (Hypothalamic-pituitary-adrenal axis) axis will be activated adult male Sprague-Dawley rats [22]. Nevertheless, this effect was mostly seen in acute alcohol administration. However, in chronic ethanol stress, tolerance will occur in the HPA axis that may incline individuals to relapse and heavy drinking [23]. This relapse results mainly through exacerbation of the existing ethanol withdrawal signs in the mouse model [24]. Other studies with alcohol proposed a role for GABAergic receptors in rodents and humans [25]. An experiment for this purpose showed antagonist of the glucocorticoid receptor can precipitate drug-seeking behavior in a stress state in a study with psychostimulant drugs and high alcohol drinkers [26, 27] but in alcohol-dependent, individuals reverse occurred [28]. Also, there is another mechanism for relapse that is due to neuroadaptation as the result of the compensatory rise in stress hormones in chronic abusers that predisposes individuals to relapse to drug-seeking behavior in studies with alcohol and cocaine in rodent studies [29–31]. In this sense, a wide variety of differences among individuals as the result of different region dependent-receptors expressions in related brain areas exists that were obtained by rodent studies [32]. In a recent study prefrontal cortex has been shown to be important in this kind of relapse in different studies in animals and human subjects [33]. Also, epigenetic changes in glucocorticoid receptors in related brain areas change addictive behavior that was observed in clinical studies [34, 35]. Epigenetic changes are described as the changes that occur in gene expression as the result of methylation or acetylation without affecting gene sequences. Epigenetic changes are a new interesting topic in this sense and are well described in different studies [36].

## 4. Sex hormones

A growing body of evidence suggests that sex hormones are strong mediators of addiction propensity [6, 37]. In animal studies, male rats showed alcohol-dependence quicker than female rats and also recover slower than male rats [38]. Differences in consumption of alcohol were observed that was lower in male compared to female in adolescent and adult Sprague-Dawley rats [39]. Also, sex hormones influence the acquisition, maintenance, and reinstatement that was well observed both in human and rodent studies[40]. In human studies, there are several variations among individuals. Overall women abusers are fewer than men abusers. Meanwhile, it should be considered that this may be dependent on opportunity and chance of access that is occurred as the result of the sex hormones influences that were observed in human studies [41, 42]. On the other hand, women tend to increase the rate of consumption of addictive substances more rapidly than men [43]. Because higher reactivity of women to stress, women find quitting more difficult than men [34]. This difference may be reflected in any stages of addition progression such as acquisition, maintenance, dysregulation-escalation, and relapse. There is evidence that estradiol has a protective role in smoking cessation in women [44]. It is thought estradiol reduces anxiety and enhances positive effects. There is strong evidence that sex hormones influence reward center maturation and efficacy that are well observed in rodent and human studies [45, 46]. However, about the application of testosterone in individuals with substance use disorder further studies are needed. More studies should be done to answer the question that what is the molecular mechanism behind this phenomenon. Based on recent studies, addictive drugs influence different sexes differently that are well documented in different types of human and rodent studies. Based on this finding it can be considered that there is a wide difference among individuals to react to addictive drugs [40].

## 5. Thyroid hormone

Thyroid hormones are essential hormones that are needed for normal bodily functions. Thyroid dysfunction is a common finding in alcoholism. Subclinical and clinical hypothyroidism may result in the development of depression and cognitive impairment. These cognitive and mood abnormalities increase the risk of relapse to addiction. It should be noted that there are great differences among individuals in this sense [47]. Although there are great controversies about the TSH (thyroid-stimulating hormone) changes in addiction, several studies indicate TSH changes that result in the alternation of T3 and T4 although there are studies that there has been an alternation of T3 and T4 without any significant change of TSH [48]. Some studies suggest the importance of thyroid hormone replacement for successful anti-smoking treatment that results in better cognitive function that is necessary for successful treatment [49]. Some studies suggest the direct toxic effect of addictive drugs on thyroid function that result in compensatory activation of the hypothalamic-pituitary axis with an increase in TRH (thyrotrophin-releasing hormone) secretion. Thyroid hormones are known agents that are used for the improvement of affective disorders [50]. The affective disorders are prevalent in abusers of drugs. In a user of heroin, circulating total thyroxin and triiodothyronine and TBG (thyroxin binding globulin) were increased but reverse triiodothyronine was normal. Human studies also show, there was a diminished response of thyrotrophin to thyrotrophin-releasing hormone and after cessation of heroin, the abnormal levels came back to the normal state [51]. In a narcotic study, the results were slightly different and it was seen that there was a slight increase in total T3 and a decrease in T3RU, and serum-free T4 level was decreased [48]. The decrease in thyroid hormone in drug users may suggest replacement therapy may reverse some aspects of drug side-effects that may interfere with successful treatment especially in heavy users [52]. Animal studies on this topic are limited.

## 6. Adipocyte-derived hormones and other nutritional regulators

Drug addiction is associated with many pathophysiological problems that result in disturbances of nutritional status [53]. Drug addicts usually suffer from malnutrition that often is associated with a lack of food availability [54]. The changes in nutritional intake result in adipose tissue-derived hormones and immune disorders that complicate methadone therapy. In an animal study with methadone, there was a significant abnormality in glucose and lipid profile [55]. The nutritional imbalances may negatively impair methadone therapy in drug abusers. Adipose tissue-derived hormones like leptin, adiponectin, and resistin are hormones that regulate the metabolic state of individuals [56–58]. It is suggested that a far better balance of those hormones helps a person with a drug use disorder to better combat the addiction. In a recent study, it has been shown that in heroin users, basal serum leptin and adiponectin levels were significantly decreased while serum resistin concentrations were raised compared to healthy subjects. It has been suggested that these changes are independent of nutritional status and insulin sensitivity among ‘individuals’ [53]. Another hormone in this topic is α-MSH one of the hypothalamic melanocortins that regulate food intake [59]. This hormone complicated the opiate maintenance program. This hormone mainly uses for food-craving. α-MSH has an antagonistic effect compared to opioids at many loci including the hypothalamus. Similarities between drug and eating addictions have been noted in recent studies. This hormone has recently got a great interest in getting better insight into opioids craving [59].

## 7. Methadone therapy and its complications

Besides the importance of the above hormones in the treatment of untreated addiction, it would be helpful for establishing an effective treatment during methadone therapy, hormonal deregulations as the consequence of methadone therapy to be considered [60, 61]. After methadone therapy, a wide variety of hormonal imbalances develops that has been well observed in human studies. These are included hypogonadism [62], sexual dysfunction [63, 64], decreased testosterone in serum [65], hyperprolactinemia [66], hypercalcitonemia [67], reduction of thyroid hormone secretion, and TSH (Thyroid-stimulating hormone) [68], adverse effect in pregnancy [69], and decreased bone density [70]. Disrespecting these abnormalities during methadone therapy may result in difficulties in reaching a successful treatment. Also, this misunderstanding may cause people with a drug use disorder to relapse to drug use for preventing the concurrence of such undesired side-effects. Some clinical studies have recommended patients may get benefit from hormonal replacement therapy, especially for sexual dysfunction [62]. However, some other clinical studies suggest the application of buprenorphine as the replacement therapy of methadone may reduce the side-effects [71].

## 8. Conclusion

Overall, it can be concluded that neurohypophyseal hormones, stress hormones, and sex hormones can interfere with tolerance, sensitization, and binge intake. About other hormones such as thyroid hormones, more studies should be done to reveal the different aspects of them in different contexts that improve high-risk behavior. Adipocyte-derived hormones seem to have a protective effect in the abstinence period especially in heroin users by improving the nutritional status of the individuals. Also, different hormonal imbalances during methadone therapy may discourage patients to deal with treatment-related problems that are most relevant to the specific hormonal imbalances that are all mentioned. Therefore, some suggest replacement therapy with hormones that are mentioned to be imbalanced during methadone therapy may encourage individuals to continue treatment. It is possible by considering the above findings more purposeful and promising treatment for patients with a drug use disorder implemented. Also, the level of hormones may predict the outcome of addiction.
